# A Professional Basketball Player Who Suffered an Open Ankle Dislocation Without an Associated Fracture Achieves His Prior Performance Level Three Months Later

**DOI:** 10.7759/cureus.64314

**Published:** 2024-07-11

**Authors:** Tatsuru Sonobe, Kazuyuki Watanabe, Yasuhiro Endo, Takuya Nikaido, Yoshihiro Matsumoto

**Affiliations:** 1 Department of Orthopaedic Surgery, Fukushima Medical University, Fukushima, JPN; 2 Department of Research for Spine and Spinal Surgery, Fukushima Medical University, Fukushima, JPN; 3 Department of Physical Therapy, Fukushima Medical University, Fukushima, JPN

**Keywords:** early return without complications, early rehabilitation with weight-bearing, conservative therapy, professional basketball player, pure open ankle dislocation

## Abstract

An ankle dislocation without an accompanying fracture is extremely rare, and an open ankle dislocation is even rarer. Due to its rarity, there is no consensus on the optimal treatment strategy. A professional basketball player (a 28-year-old male) incurred an open ankle dislocation (with no accompanying fracture) during a basketball game due to plantar flexion and inversion of his ankle during the transition from dashing to stop motion. The same day, an emergency reduction under spinal anesthesia was performed with primary closure of the wound. Considering the complications of infection and decreased ankle range of motion (ROM), primary ligament repair was not performed. He was treated conservatively with cast immobilization for four weeks, and early weight-bearing and ROM exercises were initiated. At six weeks postoperatively, stress radiography did not reveal ankle instability. After three months of conservative treatment, the patient was able to play basketball at his previous performance level. Four weeks of cast immobilization without ligament repair plus early rehabilitation with weight-bearing and ROM exercises allowed for an early return without complications. Even in high-level athletes, open ankle dislocation without an accompanying fracture can be treated adequately with conservative therapy.

## Introduction

The occurrence of an ankle dislocation without an accompanying fracture is an extremely rare injury, accounting for only 0.065% of all ankle injuries and 0.46% of all ankle dislocations [1,2]. The relative weakness of the bones compared to the ankle's mortise and the resistance of the ankle ligaments contribute to ankle dislocations, which are usually accompanied by a lateral, medial, or posterior malleolar fracture [3]. This condition is thus referred to as "pure dislocation." Pure dislocations usually occur in the setting of high-energy trauma, such as a motor vehicle accident or sports injuries [4]. Most pure ankle dislocations are closed dislocations [5]; open dislocations are extremely rare [6]. Several studies have described the treatment of an open ankle dislocation without an accompanying fracture with an external fixator [4,6,7] and/or ligament repair [6] to achieve stability; however, due to the rarity of such injuries, a standard treatment protocol has not been established.

We describe the case of an open pure ankle dislocation in a professional basketball player. The patient received conservative treatment and was able to return to playing basketball at his previous performance level without complications of pain or limited range of motion (ROM) within three months after the injury.

## Case presentation

The 28-year-old man was injured due to plantar flexion and inversion of his right ankle during the transition from dashing to stop motion while he was playing basketball. He was immediately transported to our hospital.

**Figure 1:**
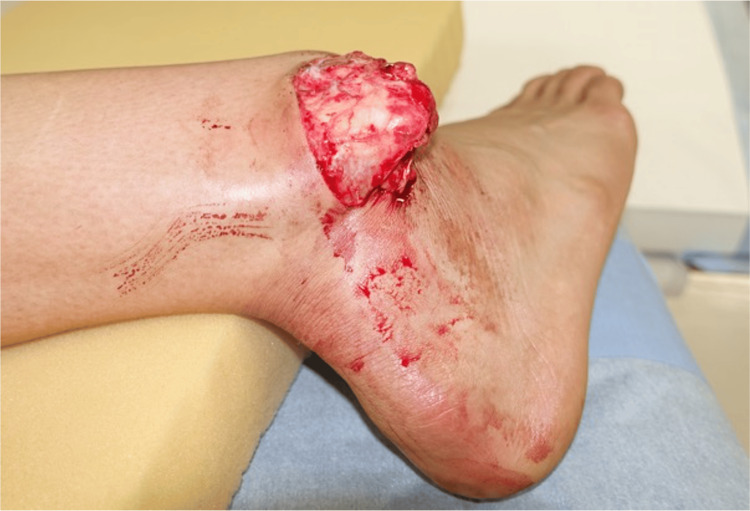
The patient's wound at the time of injury. The right fibula is exposed through the wound.

At the time of the patient's initial examination, his right ankle had deformities and was swollen. There was an open, approximately 5 cm transverse wound on the lateral side of the right ankle, with the tip of the fibula exposed through the wound (Figure 1).

**Figure 2:**
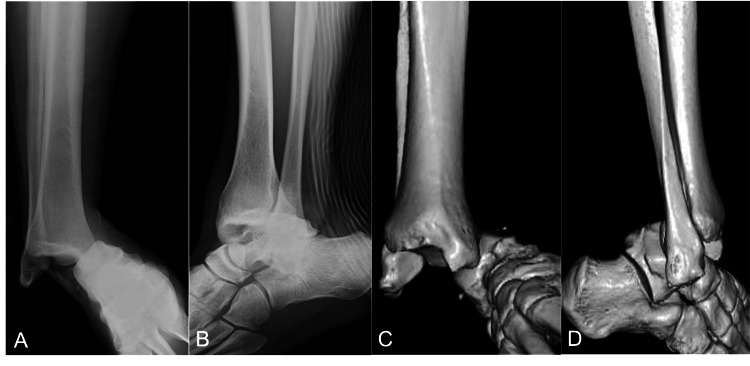
Preoperative plain radiographs and CT scans of the patient's right ankle. Plain anteroposterior (A) and lateral (B) radiographs revealed dislocation. The 3D-CT scans (C, D) showed dislocation without any associated fracture.

There was no sensory disturbance, and the dorsal foot and posterior tibial arteries were palpable. Plain radiographs and plain computed tomography (CT) revealed a posterior medial dislocation of the right ankle without any fracture (Figures 2A-D).

**Figure 3:**
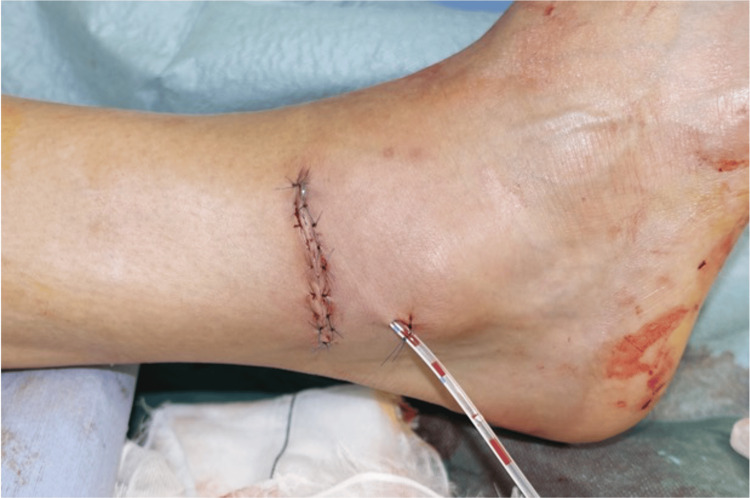
The postsurgery status of the wound. The wound was completely closed, and a drain was placed.

The injury was diagnosed as an open ankle dislocation without an associated fracture. Emergency surgery under lumbar anesthesia was performed on the day of the injury. The intraoperative findings revealed tears in the anterior joint capsule and lateral ankle ligaments. Grossly, cartilage damage to the talus was not evident. The reduction was obtained by longitudinal traction and eversion of the ankle. The wound was washed and then closed with a drain placed without a repair of the joint capsule or ligaments (Figure 3).

**Figure 4:**
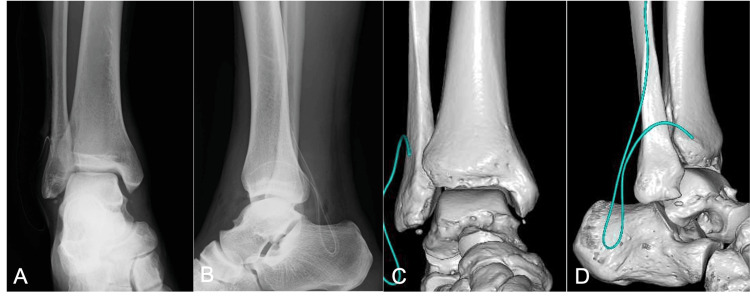
Postoperative plain radiographs and CT scans of the patient's right ankle. Plain anteroposterior (A) and lateral (B) radiographs revealed reduction. The 3D-CT scans (C, D) showed a small bone fragment at the tip of the fibula, which was thought to be an old avulsion fracture.

Postoperatively, no sensory or circulatory disturbances were observed. After the reduction, the patient's ankle was immobilized in a short leg cast. The plain radiographs and CT scans showed no evidence of bone fracture (Figure 4).

**Figure 5:**
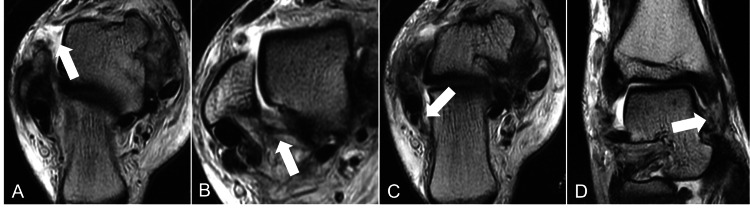
Postsurgery T2-weighted MRI of the patient's ankle. Axial T2-weighted MRI of the right ankle revealed tears in the anterior talofibular ligament (ATFL) (A), posterior talofibular ligament (PTFL) (B), and calcaneofibular ligament (CFL) (C) (arrow). The deltoid ligament (DL) (D) was indistinct in the deep layers, but the shallow layers remained (arrow). No cartilage damage to the talus was observed.

A small bone fragment was seen on the distal fibula, but the margins were sclerotic, suggesting an old avulsion fracture. T2-weighted magnetic resonance imaging (MRI) revealed tears in the anterior joint capsule, anterior talofibular ligament (ATFL) (Figure 5A), posterior talofibular ligament (PTFL) (Figure 5B), and calcaneofibular ligament (CFL) (Figure 5C). The deltoid ligament (DL) was indistinct in the deep layers, but the shallow layers remained. No cartilage damage to the talus was observed (Figure 5D).

The patient was discharged two weeks after the surgery when the swelling and pain were under control, and the wound had healed. Cast immobilization and non-weight-bearing status were applied for four weeks postsurgery. Four weeks after the surgery, the cast was removed, and an ankle brace was used for ROM exercises in plantar dorsiflexion while inversion and eversion were restrained. ROM exercises for the ankle were begun, and full weight-bearing was allowed without restrictions. At this point, the patient reported no pain during weight-bearing. The passive ROM of the right ankle was 5° dorsiflexion and 35° plantar flexion.

**Figure 6:**
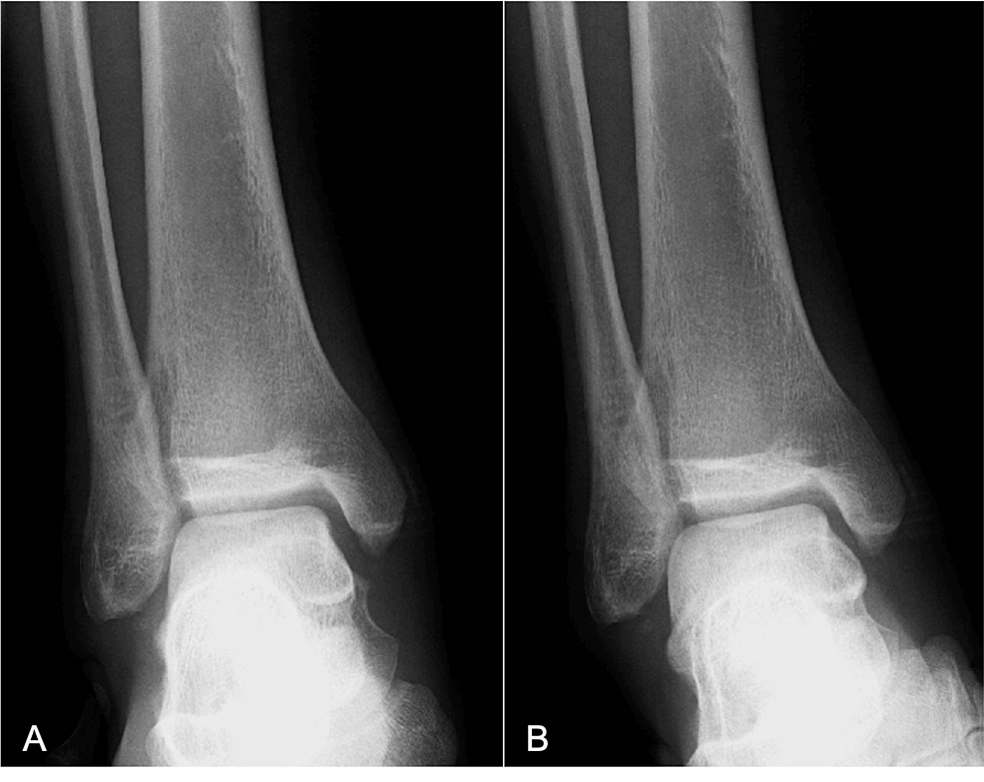
Stress radiography of the right ankle at six weeks postsurgery. The neutral position (A) and inversion stress (B) revealed no ankle instability.

At six weeks postsurgery, instability of the right ankle was evaluated by stress radiography (neutral position: Figure 6A, inversion stress: Figure 6B).

The talar tilt angle by inversion stress was 10°, which did not meet the criterion for instability (≥15°) [8]. The patient's ankle brace was switched to a supporter in order to prevent swaying during landing, and the patient began jogging, calf raises, and bipedal jumps. At this point, the passive ROM of the right ankle was 15° dorsiflexion and 45° plantar flexion. At eight weeks postsurgery, rehabilitation was allowed with no restrictions, and the patient began sidestepping, pivoting movements, and 50% dash. The passive ROM of his right ankle improved to 20° dorsiflexion and 50° plantar flexion, and the left-right difference disappeared.

**Figure 7:**
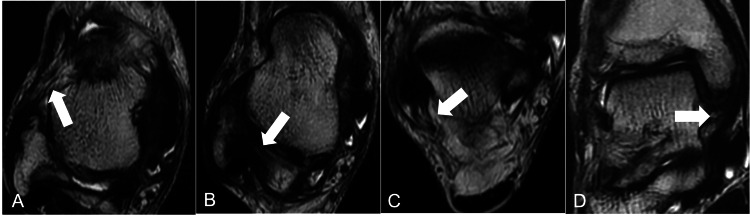
T2-weighted MRI of the patient's ankle at 11 weeks postsurgery. Axial T2-weighted MRI of the right ankle indicated scarring at the ATFL tear (A) (arrow). The PTFL (B) and CFL (C) were spontaneously repaired. The running of the DL (D) was clarified, and the tension was improved (arrow).

After 10 weeks postsurgery, the patient was allowed to engage in full-strength dashes, side jumps, and lay-up shooting. A follow-up MRI examination was performed 11 weeks postsurgery, which visualized scarring at the ATFL tear (Figure 7A) and revealed that the PTFL (Figure 7B), CFL (Figure 7C), and DL (Figure 7D) had spontaneously repaired. Although mild swelling of the right ankle remained, the patient reported no ankle pain.

At 11 weeks postsurgery, a physical examination confirmed that the ROM was normal and there was no instability. The patient was allowed to play basketball without limitations (including contact play). At 12 weeks after the injury, he returned to playing in the Japan Professional Basketball League in the same season.

During the three-month follow-up examination, the American Orthopaedic Foot and Ankle Society Ankle-Hindfoot Score (AOFAS-AHS) [9] of the patient was good at 92 points. He described being able to play basketball with no limitations. He played as a starter in 22 of 25 games. Since his return to the game, he played an average of 20.7 minutes, with an average of 3.11 points, 1.09 assists, and 2.73 rebounds (before the injury, his average playing time was 20.6 minutes with an average of 4.5 points, 2.0 assists, and 3.83 rebounds).

## Discussion

Ankle sprains have been described as the most common injury sustained by basketball players [10]. Lateral ligament sprains are the most common, followed by DL injuries [10]. Other foot and ankle joint injuries that occur in competitive basketball players include lateral ligament rupture, Achilles tendon rupture, plantar fasciitis, fifth metatarsal fracture [10], and rarely, ankle dislocation [6].

Pure ankle dislocations are rare, and the relevant literature remains limited [1-7,11-15]. Open dislocations are particularly rare [6]. The mechanism of injury is thought to be axial loading, with the ankle landing in plantar flexion and adduction [11]. The present patient was injured while engaging in a full-speed dash, catching up to the opposing player, and then moving into a defensive action. We speculate that the injury was caused by plantar flexion and inversion of his ankle during the transition from dashing to the stop motion.

The etiology of pure ankle dislocations includes medial malleolus hypoplasia, ligament laxity, weakness of the peroneal muscles, and repetitive ankle sprains [16]. In their assessment of internal malleolus hypoplasia, Elisé et al. reported that with the distance from the tibial canopy to the tip of the lateral malleolus defined as "A" and the distance to the medial malleolus defined as "B" on anteroposterior ankle radiographs, a normal B/A value is 0.58-0.62 [17]. In our patient's case, there was no medial malleolus hypoplasia, as the value was 0.608. However, he reported a history of frequent ankle sprains and was aware of internal instability, which may have been a predisposing factor for the present injury.

In 2014, Bhullar et al. noted that neurovascular compromise prior to reduction occurred in 19% of pure ankle dislocations [1]. There is no dispute that an immediate reduction is necessary to reduce the risk of neurovascular complications. In addition, prompt intravenous antibiotic prophylaxis is recommended because of the risk of infection in an open injury [12]. Our patient did not develop any neurovascular complications or infection due to the prompt intravenous antibiotic prophylaxis and washout of the wound on the day of his injury. While there is consensus regarding the initial treatment described above, there is controversy regarding the conservative treatment method and the necessity of surgical repair of the injured ligament.

For conservative treatment, short leg casts or external fixators have been used [2,3,7]. No instability was reported in patients treated with cast immobilization [13]. The use of an external fixator to stabilize the ankle and facilitate wound care has been proposed [7] since infection is a concern in cases of open dislocation [6]. The use of an external fixator is an easy approach to obtaining firm fixation, but there are concerns about pin-site infection and decreased ROM due to firm fixation [7]. Our patient was treated with cast immobilization because the wound was in good condition, and the cast provided stability.

Regarding limitations of weight-bearing for the conservative treatment of an ankle dislocation, the reported average postsurgery time to allow full weight-bearing is 6.2 weeks [14], although other researchers recommend early weight-bearing [11] because a longer period of immobilization enhances the stiffness of the ankle [18]. No pain or instability was described in a case with a four-week cast fixation period [18]. Since our patient was a professional basketball player, the ankle contracture is directly related to his athletic performance. To avoid decreased ankle ROM, we set his cast immobilization period at four weeks, and full weight-bearing was started sooner than the reported average, i.e., 6.2 weeks [14]. Ankle instability was not observed with the shortening of the immobilization period and the early initiation of full weight-bearing. Even after the patient's cast mobilization was finished, the use of a brace to restrict inversion and eversion may have contributed to the acquisition of ankle stability. As in an earlier case involving an athlete [18], we inferred that a cast fixation period of four weeks was sufficient.

Regarding torn ligaments, the necessity of surgical repair is controversial. It was reported that 46% of patients with ankle joint dislocations received nonsurgical treatment, while only 26% underwent ligament repair [2]. Good clinical outcomes without repair of the ankle ligaments have been described [2]. Conversely, cases with no repair of lateral ligaments showed moderate ankle instability, followed by the development of degenerative changes [15]. For open dislocations, the repair of torn ligaments has been recommended by some authors [2,14], while others reported good long-term results without ligament or capsule repair [12,14]. Particularly, in cases of complete lateral ankle ligament tears, a short period of immobilization followed by ROM exercises has provided outcomes similar to those of ligament repair [19]. Primary ligament repair was not performed in the present patient on the same day of his injury due to concerns about infection and contracture, and a planned ligament repair was not performed because of concerns about ankle contraction; conservative treatment was chosen. Early rehabilitation was adopted to reduce the patient's ankle contracture as much as possible, and ligament repair was considered when instability remained. The patient had no ankle instability in stress radiography at six weeks postsurgery, indicating that he could continue to be treated conservatively. Surgical ligament repair may be optimal in patients for whom conservative treatment has not been successful and instability remains.

The most common complication described in the literature is a decrease in the ROM [2]. In professional basketball players, decreased ROM of the ankle is a career-ending condition. We minimized the duration of the present patient's cast immobilization in order to prevent contraction, and weight-bearing and ROM exercises were initiated early with an ankle brace to gain ankle stability. Early weight-bearing and active-controlled motion in rehabilitation have been shown to improve outcomes for ankle fractures, enhancing ROM and stability [20]. Our patient was provided a menu of weight-bearing exercises and instructions to engage in the exercises as much as possible during rehabilitation. The intensity of the rehabilitation menu was increased according to the patient's degree of edema, and the gradual acquisition of ankle ROM is thought to have contributed to his early return to playing basketball.

The patient suffered a rare open pure ankle dislocation. Even in a high-level athlete, four weeks of cast immobilization without ligament repair and early rehabilitation with weight-bearing and ROM exercises allowed for an early return without complications. Our patient's outcome was as good as those of basketball players with pure ankle dislocation who underwent ligament repair (AOFAS-AHS 98 points) or conservative treatment with six weeks of cast immobilization (AOFAS-AHS 100 points) [7]. A continued follow-up is planned to observe the potential for future instability and degenerative changes.

## Conclusions

A professional basketball player incurred an open ankle dislocation without an associated fracture. Conservative treatment with early weight-bearing and ROM exercises resulted in the achievement of his previous performance level three months after the injury without complications. Even in high-level athletes, open ankle dislocation could be treated conservatively with intensive rehabilitation, which consisted of early weight-bearing and ROM exercises with an ankle brace after four weeks of cast immobilization.
